# Safety and Efficacy of Aerobic Exercise Training Associated to Non-Invasive Ventilation in Patients with Acute Heart Failure

**DOI:** 10.5935/abc.20180039

**Published:** 2018-05

**Authors:** Mayron F. Oliveira, Rita C. Santos, Suellen A. Artz, Vanessa M. F. Mendez, Denise M. L. Lobo, Edileide B. Correia, Almir S. Ferraz, Iracema I. K. Umeda, Priscila A. Sperandio

**Affiliations:** 1 Instituto Dante Pazzanese de Cardiologia, São Paulo, SP - Brazil; 2 Universidade de Fortaleza (UNIFOR) - Centro de Ciências da Saúde, Fortaleza, CE - Brazil; 3 Faculdade Metropolitana da Grande Fortaleza (FAMETRO), Fortaleza, CE - Brazil

**Keywords:** Exercise, Acute Heart Failure, Non-Invasive Ventilation, Physiotherapy, Rehabilitation

## Abstract

**Background:**

Exercise training (ET) improves functional capacity in chronic heart failure (HF). However, ET effects in acute HF are unknown.

**Objective:**

To investigate the effects of ET alone or combined with noninvasive ventilation (NIV) compared with standard medical treatment during hospitalization in acute HF patients.

**Methods:**

Twenty-nine patients (systolic HF) were randomized into three groups: control (Control - only standard medical treatment); ET with placebo NIV (ET+Sham) and ET+NIV (NIV with 14 and 8 cmH_2_O of inspiratory and expiratory pressure, respectively). The 6MWT was performed on day 1 and day 10 of hospitalization and the ET was performed on an unloaded cycle ergometer until patients' tolerance limit (20 min or less) for eight consecutive days. For all analyses, statistical significance was set at 5% (p < 0.05).

**Results:**

None of the patients in either exercise groups had adverse events or required exercise interruption. The 6MWT distance was greater in ET+NIV (Δ120 ± 72 m) than in ET+Sham (Δ73 ± 26 m) and Control (Δ45 ± 32 m; p < 0.05). Total exercise time was greater (128 ± 10 vs. 92 ± 8 min; p < 0.05) and dyspnea was lower (3 ± 1 vs. 4 ± 1; p < 0.05) in ET+NIV than ET+Sham. The ET+NIV group had a shorter hospital stay (17 ± 10 days) than ET+Sham (23 ± 8 days) and Control (39 ± 15 days) groups (p < 0.05). Total exercise time in ET+Sham and ET+NIV had significant correlation with length of hospital stay (r = -0.75; p = 0.01).

**Conclusion:**

Exercise training in acute HF was safe, had no adverse events and, when combined with NIV, improved 6MWT and reduce dyspnea and length of stay.

## Introduction

Heart failure (HF) is a complex syndrome characterized by reduced left ventricular function, skeletal myopathy and exercise intolerance.^1^^,^^2^ Previous studies have shown evidences that exercise training (ET) can be an effective non-pharmacological intervention for patients with chronic HF.^3^^-^^6^ However, periods of acute/decompensated HF may occur, which represent the most frequent cause of hospitalization,^7^ leading to long periods of bed rest and sarcopenia^8^^,^^9^ and, consequently, complications during hospitalization.

Acute HF patients show worsening pulmonary congestion, dyspnea, increased respiratory effort, exercise intolerance^10^ and frequently, decreased alveolar ventilation, which results in blood shunting and hypoxemia.^11^ In this context, noninvasive ventilation (NIV) has been widely used in acute HF cases to reduce dyspnea and improve oxygenation.^12^^,^^13^

In addition, patients with chronic HF have displayed a progressive reduction in functional capacity and decreased exercise tolerance compared to healthy individuals, due to both cardiac disease and peripheral factors (endothelial dysfunction, inflammation, and increased neurohormonal activation).^14^^,^^15^ Moreover, it has been already demonstrated that exercise with NIV in chronic HF increases exercise tolerance and reduces dyspnea and leg effort.^16^^,^^17^

Several studies have shown that early exercise after admission can benefit critical patients in the intensive care unit^18^^,^^19^ and patients with chronic obstructive pulmonary disease exacerbations.^20^^,^^21^ These studies showed a reduction in length of stay and rehospitalization, as well as improved quality of life. However, ET in acute HF patients has been contraindicated and there have been no studies to evaluate the effects of cardiac rehabilitation on acute/decompensated HF. Thus, despite extensively documented evidence regarding the benefits of exercise^5^^,^^22^ and NIV combined with exercise^16^ in chronic HF patients, the safety and effectiveness of aerobic ET in acute HF patients remains unknown.

Therefore, in the present study, we aimed to investigate in acute/decompensated HF patients, (i) the safety of in-hospital aerobic ET; and (ii) the effectiveness of aerobic ET combined with NIV during hospitalization in patients with acute HF.

## Methods

This was a controlled, prospective and randomized study. A convenience sample of 29 patients was recruited from the acute HF ward of a cardiology hospital. These patients had an established diagnosis of acute HF and a previous Doppler echocardiography with left ventricle ejection fraction (LVEF) < 30%. All of them were in NYHA class IV.

Patients were excluded from the study if they had unstable angina, complex cardiac arrhythmias, pacemaker, cardiac resynchronization therapy or left ventricular assist device, myocardial infarction within the previous 12 months, oxyhemoglobin saturation by pulse oximetry (SpO_2_) at rest <88% without oxygen supplementation, or acute pulmonary edema with clinical indications for mechanical ventilation. In addition, patients with clinical indication of NIV besides the proposed by this protocol were excluded.

### Study protocol

All of the subjects underwent an individualized clinical evaluation after hospital admission on day 1 (D1) by the cardiologist and physiotherapist involved in the study. Pulmonary function tests (spirometry), blood sample (brain natriuretic peptide [NT-proBNP] and high sensitivity C-reactive protein [hs-CRP]), six-minute walk test (6MWT), and maximal inspiratory pressure (MIP) test were performed. 

All patients received standard medical treatment^7^ and after clinical and laboratorial tests they were randomized into three groups: ET+NIV, ET+Sham and Control. We decided to include a placebo NIV group to test the hypothesis that exercise alone (ET+Sham) or exercise associated to NIV (ET+NIV) were better than conventional treatment (Control group) in acute heart failure patients.

The ET+NIV group performed aerobic ET associated with NIV once a day, for 8 consecutive days; and the ET+Sham group performed aerobic exercise with placebo NIV once a day, also for 8 consecutive days. The control group (Control) received only medical treatment and did not perform aerobic exercise training.

At D10 all patients underwent the same clinical evaluation as D1. After the protocol, all patients continued receiving only medical treatment, and were followed-up until hospital discharge or transfer to the intensive care unit.

### Exercise protocol

The ET+NIV and ET+Sham groups performed aerobic exercise on an unloaded in-bed cycle ergometer (Cajumoro, Brazil) for 20 minutes or less, until limit of tolerance. The exercise groups were blinded to pressure applied to NIV or Sham. SpO_2_ (Nonin^®^ Medical, USA) and heart rate (HR) were continuously measured with a heart rate monitor (Polar^®^ RS800, Finland). Systolic and diastolic arterial pressures (SAP and DAP) were obtained by the auscultatory method (Unilec^TM^ sphygmomanometer and Littmann Quality stethoscope; USA). Blood lactate (Accutrend Plus^®^, Germany) was collected during the exercise protocol, at rest, every two minutes, and at the end of exercise. The patients were asked to rate their "shortness of breath" at exercise cessation by the 0-10 Borg's category ratio scale.^23^

### Noninvasive positive pressure ventilation

Noninvasive ventilation was delivered using the bi-level ventilator (BiPAP Vision^®^; Respironics, USA), applied via oronasal mask in two conditions: bi-level positive airway pressure ventilation - inspiratory positive airway pressure: 14 cmH_2_O, and expiratory positive airway pressure: 8 cmH_2_O, without supplementary oxygen (FiO_2_ 0.21) and sham ventilation - inspiratory positive airway pressure: 4 cmH_2_O, and expiratory positive airway pressure: 4 cmH_2_O, without supplementary oxygen (FiO_2_ 0.21).

The pressure values were selected based on previous evidence that an inspiratory positive pressure range of 8-20 cmH_2_O and a positive end-expiratory pressure range of 4-10 cmH_2_O were associated with positive clinical effects in a population with similar levels of acute HF.^24^^,^^25^ The inspiratory positive pressure and positive end-expiratory pressure values in the sham NIV were set to minimum value (4 cmH_2_O), since the BiPAP Vision cannot be reduced below 4 cmH_2_O. Those values in sham NIV were able to overcome the resistance imposed by the ventilator circuit (as directed by the manufacturer) and to ensure that patients remained blinded to the intervention being applied.

### Pulmonary function test and maximal inspiratory pressure

Spirometric tests were performed, and forced expiratory volume in 1 second (FEV_1_), forced vital capacity (FVC), and FEV_1_/FVC ratio were measured (EasyOne^®^ Plus Diagnostic spirometer, Switzerland). 

MIP was measured with a digital manometer (MVD-300^®^ V.1.1 Microhard System; Globalmed, Brazil). Patients were instructed to perform a maximum inspiration from residual volume; each patient performed five maximum inspirations with differences smaller than 10% between them, and the highest result was used for the analysis. Therefore, all results were compared to predicted values.^26^

### Six-minute walk test

The 6MWT was performed on a 30-m flat corridor, according to the American Thoracic Society.^27^ Blood pressure, HR, and SpO_2_ were measured, and the modified dyspnea Borg scale was applied. All measurements were performed before and immediately after completion of the tests, and after a two-minute recovery period. HR and SpO_2_ were monitored throughout the test (Nonin^TM^ portable oximeter - USA).

### Statistical analysis

Statistical analysis was carried out with the SPSS software (version 20.0, SPSS Inc., USA). Data were expressed as mean ± standard deviation or as median and interquartile range, as appropriate, and categorical data are expressed as frequency (n and %). The normality of data distribution was determined by Shapiro-Wilk test. The chi-square test was used to assess differences between categorical data, and repeated-measures ANOVA followed by Bonferroni corrections were used for multiple comparisons. Pearson's correlation was used for parametric correlations. For all analyses, statistical significance was set at 5% (p < 0.05).

## Results

### Baseline measures

Twenty-nine patients who fulfilled all the inclusion criteria were enrolled in the study and randomized into three groups: Control (n = 9, 58 ± 7 years of age), ET+Sham (n = 9, 57 ± 5 years) and ET+NIV (n = 11, 56 ± 8 years). All patients had diagnosis of acute HF. There were no differences in anthropometric and demographic variables, cause of HF, LVEF, main comorbidities, medications, and NT-proBNP or hs-CRP plasma levels among groups (Table 1). The functional class, exercise tolerance and pulmonary function were not different among groups (Table 2).

**Table 1:** Baseline characteristics of hospitalized acute heart failure patients allocated into one of the three groups - exercise training + non‑invasive ventilation (ET+NIV), ET + Sham or Control group

	Control (n = 9)	ET+Sham (n = 9)	ET+NIV (n = 11)
**Anthropometrics/Demographics**			
Male, n (%)	7 (78%)	8 (89%)	7 (64%)
Age, years	58 ± 7	57 ± 5	56 ± 8
Weight, kg	65.3 ± 14.8	74.0 ± 13.5	66.4 ± 10.8
Height, m	1.60 ± 0.71	1.68 ± 0.10	1.64 ± 0.40
BMI, kg/m^2^	24.2 ± 5.0	26.9 ± 4.6	24.8 ± 4.0
LVEF, %	23.8 ± 4.9	25.4 ± 6.7	26.0 ± 4.8
NTpro-BNP, ρg/mL	2467 ± 547	2331 ± 429	2594 ± 633
hs-CRP, mg/L	8 ± 3	9 ± 4	9 ± 5
Length of stay, days	39 ± 15	23 ± 8*	17 ± 10*^†^
**Main comorbidities**			
Hypertension, n (%)	5 (56%)	3 (33%)	5 (54%)
Dyslipidemia, n (%)	4 (44%)	1 (11%)	1 (9%)
Diabetes mellitus, n (%)	2 (22%)	2 (22%)	1 (9%)
**Etiology**			
Ischemic, n (%)	6 (67%)	7 (80%)	7 (44%)
**Main medications**			
β-blocker, n (%)	7 (78%)	6 (67%)	8 (73%)
ACE inhibitors or ARBs, n (%)	4 (43%)	6 (63%)	7 (64%)
Diuretics, n (%)	9 (100%)	9 (100%)	11 (100%)

Definition of abbreviations: BMI: body mass index; LVEF: left ventricular ejection fraction; NTpro-BNP: brain natriuretic peptide; hs-CPR: high sensitive C-reactive protein; ACE: angiotensin conversor enzyme; ARBs: angiotensin II receptor blockers. Values expressed as mean ± standard deviation or frequency (n and %). Repeated-measures ANOVA with appropriate Bonferroni corrections was applied to variables described as mean ± standard deviation and the chi-square test was used to assess differences in categorical data.

* p < 0.05 vs. Control;

† p < 0.05 vs. ET+Sham

**Table 2:** Characteristics of the "exercise training + non-invasive ventilation (ET + NIV)", "ET + Sham" and Control groups at hospital admission and after study protocol

	Day 1			Day 10		
	Control	ET+Sham	ET+NIV	Control	ET+Sham	ET+NIV
**NYHA**						
II, n (%)	-	-	-	3 (33%)	5 (55%)*	8 (72%)*
III, n (%)	-	-	-	4 (44%)	3 (33%)*	2 (18%)*
IV, n (%)	9 (100%)	9 (100%)	11 (100%)	2 (22%)	1 (11%)*	1 (10%)*
Dobutamine, n (%)	5 (55%)	4 (44%)	6 (54%)	3 (33%)	2 (22%)*^‡^	2 (18%)*^‡^
**Exercise tolerance**						
Total exercise time, min	-	-	-	-	92 (60 - 120)	128 (90 - 160)^†^
6MWT, m	221 ± 58	238 ± 51	224 ± 30	266 ± 83	311 ± 67*^‡^	345 ± 61*^†‡^
∆6MWT, m	-	-	-	45 ± 32	73 ± 26*	120 ± 72*^†^
**Pulmonary function**						
MIP, cmH_2_O	-65 ± 20	-53 ± 20	-60 ± 11	-64 ± 31	-61 ± 36	-63 ± 15
MIP, % predicted	73 ± 25	77 ± 33	72 ± 24	72 ± 32	75 ± 42	77 ± 22
FEV_1_, % predicted	57 ± 21	59 ± 20	61 ± 22	68 ± 29	60 ± 20	65 ± 21
FEV_1_/FVC	0.72 ± 0.18	0.79 ± 0.10	0.75 ± 0.12	0.74 ± 0.17	0.78 ± 0.18	0.76 ± 0.10

Definition of abbreviations: NYHA: New York Heart Association; 6MWT: six minute walk test; MIP: maximal inspiratory pressure; FEV1: forced expiratory volume in 1 second; FVC: forced vital capacity. Values are expressed in mean ± standard deviation; median (interquartile range) and frequency (n and %). Repeated-measures ANOVA with the appropriate Bonferroni corrections was applied to variables described as mean ± standard deviation and the chi-square test was used to assess categorical data differences in frequency variables;

* p < 0.05 vs. Control;

† p < 0.05 vs. ET+Sham;

‡ p < 0.05 vs. Day 1

### Effects of exercise training associated with NIV and sham ventilation

None of the patients of group ET+NIV or ET+Sham had any criteria for exercise interruption. Total exercise time was shorter in the ET+Sham group (~30% lower compared to ET+NIV) (Table 2).

**Figure 1:**
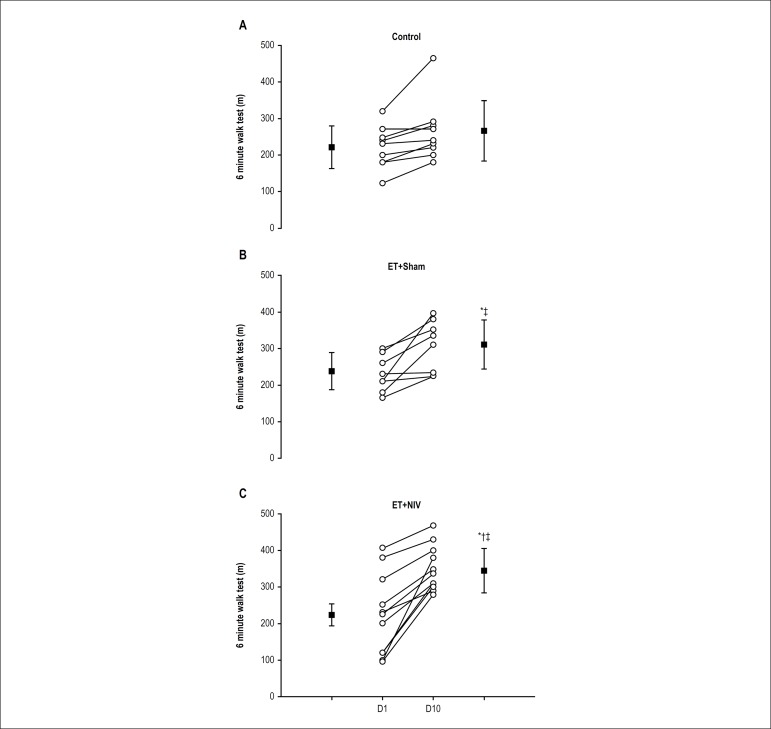
Six minute walk test distance achieved at D1 and D10 in Control, ET+Sham and ET+NIV groups. Notes: Open circles: individual distance achieved at D1 and D10. Dark square: mean and standard deviation of distance at D1 and D10. * p < 0.05 vs. Control; † p < 0.05 vs. ET+Sham; ‡ p < 0.05 vs. D1.

On D10, the ET+NIV and ET+Sham groups had a greater walking distance compared to the control group (Table 2). In addition, Δ6MWT distance on D10 was greater in the ET+NIV group (Figure 1, Panel C) than in the ET+Sham group (Figure 1, Panel B) and the control group (Figure 1, Panel A). There were no differences in blood pressure, HR and SpO_2_ during 6MWT between the groups (data not shown).

**Figure 2:**
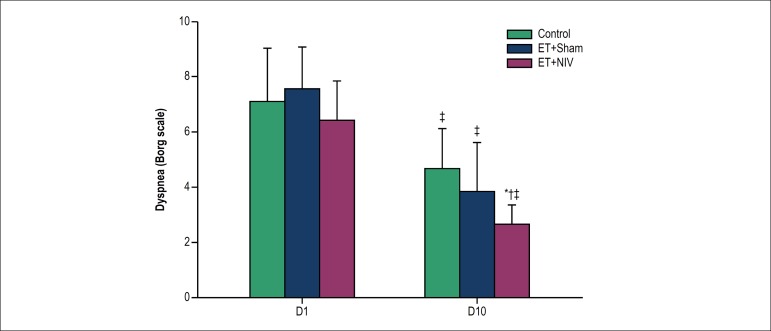
Dyspnea Borg scale at first day of hospitalization (D1) and at last day of protocol (D10) in Control, Exercise Training (ET) + Sham and ET+non-invasive ventilation (NIV) groups. Notes: * p < 0.05 vs. Control; † p < 0.05 vs. ET+Sham; ‡ p < 0.05 vs. D1.

Dyspnea score at rest was higher at baseline (D1) and decreased over time in all three groups. Moreover, ET+NIV group had the lowest dyspnea value on D10 (Figure 2). The number of patients receiving dobutamine infusion at D1 was similar among groups; however, on D10 the exercise groups (ET+Sham and ET+NIV) had a lower number of patients receiving dobutamine infusion compared to the control group (Table 2).

From D1 to D10, there was a significant reduction in NT-proBNP (ΔNT-proBNP: -892 ± 112 rg/mL [Control]; -1184 ± 299 rg/mL [ET+Sham]; -1002 ± 356 rg/mL [ET+NIV]) and hs-CRP levels (Δhs-CRP: -4 ± 2 mg/L [Control]; -4 ± 3 mg/L [ET+Sham]; -5±3 mg/L [ET+NIV]), but without differences among groups. In addition, there was a similar reduction in body weight from D1 to D10 between the three groups studied (Δweight: -3.3 ± 2.2 kg [Control]; -5.3 ± 3.9 kg [ET+Sham]; -5.0 ± 2.0 kg [ET+NIV]). No differences in small airway obstruction, MIP and blood lactate were found between the groups at D1 and at D10 (Table 2).

### Follow-up

**Figure 3:**
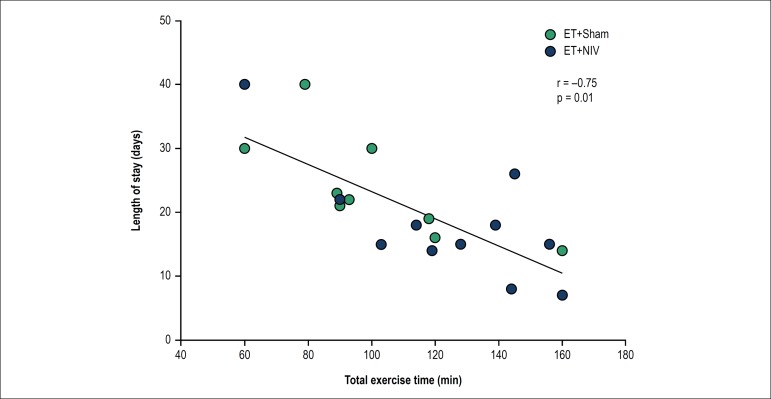
Correlation between total exercise time and length of hospital stay (days) in exercise groups.

None of the patients of the exercise groups needed to be transferred to the intensive care unit. In addition, more patients in the ET+NIV and ET+Sham groups had an early hospital discharge compared to the control group. Of note, the control group had a significantly greater length of stay compared to the exercise groups. In addition, the ET+NIV group had a shorter length of stay compared to the ET+Sham group (Table 1). Interestingly, total exercise time performed in both groups (ET+Sham and ET+NIV) was inversely related to length of stay (Figure 3).

## Discussion

To the best of our knowledge, this is the first study to assess the role of aerobic exercise training in acute/decompensated HF (NYHA class IV). The main and new findings of this study are that exercise in acute/decompensated HF (i) is safe, since neither ET+Sham nor ET+NIV groups showed worse symptoms during exercise or signs of requiring exercise interruption and (ii) reduces the length of hospital stay. In addition, the exercise increases the 6MWT distance.

Studies have demonstrated that early mobilization therapy in intensive care unit patients can significantly reduce the length of stay.^19^ It has also been demonstrated that rehabilitation immediately following an acute exacerbation of chronic obstructive pulmonary disease is associated with a reduced frequency of re-exacerbation and with an increase in quadriceps muscle strength.^20^^,^^21^ In the same line, a recently study demonstrated that functional electrical stimulation improved exercise tolerance and muscle strength in acute HF patients.^28^ Our study extends the knowledge about approaches to be used during hospitalization to treat decompensated HF patients. It suggests that aerobic exercise training *per se* is a safe and effective tool to reduce length of hospital stay in acute HF patients. It should be emphasized that none of the patients who performed exercise had worsening of symptoms during exercise or exhibited any signs of exercise intolerance.

Another important finding in our study was the increase in exercise tolerance in patients who underwent aerobic exercise. Actually, this finding has clinical implications. The 6MWT distance is associated with clinical outcome and quality of life in patients with HF.^29^ Furthermore, it is possible that the aerobic exercise training improves exercise tolerance even in hospitalized HF patients.

In order to investigate if the use of NIV could have additional effects on aerobic exercise training, we found that ET+NIV group had improved exercise performance and decreased dyspnea during the exercise. In fact, NIV might reduce venous return and cardiac preload,^30^ which could explain our findings. Another finding of great interest and clinical relevance was that the ET+NIV had a shorter hospital stay, enhanced 6MWT distance and exercise time compared to ET+Sham group, suggesting that the NIV can enhance the effectiveness of aerobic exercise in acute HF patients. The rationale for this theory is that NIV combined to exercise has some influence on redistribution of muscle blood flow.^16^ Dempsey et al.^31^ suggested that respiratory muscles influence vascular diameter and peripheral vasoconstriction. Respiratory muscles might compete for the reduced blood flow during exercise with the peripheral muscles, thereby promoting an inadequate oxygen transport and exercise fatigue. In addition, fatiguing contractions could stimulate IV phrenic afferents by muscle metabolic production, increasing sympathetic vasoconstriction with consequent reduction in oxygen delivery.^31^^,^^32^

It was recently demonstrated that patients with chronic HF has slower oxygen kinetics with increased deoxyhemoglobin kinetics during exercise.^14^ On the other hand, Borghi-Silva et al.^16^ demonstrated that NIV was able to improve exercise tolerance and reduce the deoxyhemoglobin kinetics in peripheral muscle during exercise in patients with chronic HF. In our study, the ET+NIV group showed better response to aerobic exercise. The mechanism for this response is beyond the scope of our study, however it is likely that NIV influenced muscle blood flow redistribution, from the respiratory muscles to the peripheral muscles, improving oxygen delivery and utilization.

### Study limitations

The present study has some limitations that should be addressed. First, we had a small number of patients. In addition, our patients performed an aerobic exercise without workload (unloaded exercise). We chose this type of exercise because this is the first protocol of this type in acute HF patients, and the repercussions of the exercise were unknown.

Also, we acknowledge that exercise groups performed the protocol during 8 days only, but such period was established based on the mean length of stay in our institution. Further studies on exercise and its main outcomes should be performed including the whole hospitalization period. In fact, this was the first study to perform aerobic exercise training in acute HF, so a reduced exercise protocol duration was necessary to check the viability and safety of aerobic exercise in this patient population.

Our study raises new questions regarding exercise in acute HF. Further study protocols must be performed to confirm our data, including clinical outcomes as death and worsening HF, other exercise modalities (inspiratory muscle training, resistive, etc.) and how to perform the aerobic exercise prescription in acute HF, as recently demonstrated in chronic HF.^33^

### Clinical implications

Our study provides evidences of the importance of aerobic exercise during hospitalization in acute HF patients. The findings of safety, reduced length of stay and increased exercise tolerance suggest aerobic exercise training as a new tool for the management of acute HF in combination with standard clinical therapy. Moreover, the enhance of the positive effects of aerobic exercise when combined to NIV, reinforce the relevance of our study, and opens new challenges to investigate the mechanisms of this strategy that contributes to better clinical outcomes in patients with decompensated HF.

## Conclusion

Aerobic exercise is safe, improves the exercise tolerance and reduces hospital stay for decompensated HF patients. Moreover, NIV can enhance the effectiveness of aerobic exercise in these patients. These findings suggest that this simple tool associated with standard clinical therapy may be useful during hospitalization for the management of acute HF.
